# Deubiquitinase MYSM1 Is Essential for Normal Bone Formation and Mesenchymal Stem Cell Differentiation

**DOI:** 10.1038/srep22211

**Published:** 2016-02-26

**Authors:** Ping Li, Yan-Mei Yang, Suzi Sanchez, Dian-Chao Cui, Rui-Jie Dang, Xiao-Yan Wang, Qiu-Xia Lin, Yan Wang, Changyong Wang, Da-Fu Chen, Si-Yi Chen, Xiao-Xia Jiang, Ning Wen

**Affiliations:** 1 Department of Stomatology, Chinese PLA General Hospital, 28Fuxing Road, HaidianDistrict, Beijing 100850, P.R. China; 2 Department of Advanced Interdisciplinary Studies, Institute of Basic Medical Sciences, 27 Taiping Road, HaidianDistrict, Beijing 100850, P.R. China; 3 Department of Stomatology, The 309th Hospital of Chinese People’s Liberation Army, Beijing, 100091, P.R. China; 4 Cardiovax LLC, 10100 Santa Monica Blvd, Los Angeles, California, 90067, USA; 5 Department of Anesthesiology, Beijing Aiyuhua Hospital for Children and Women, 2 South Street, Beijing economic and Technological Development Zone, Beijing 100176, P.R. China; 6 Beijing Research Institutes of Traumatology and Orthopaedics, Beijing, 100035 P.R. China; 7 Department of Molecular Microbiology and Immunology, Norris Comprehensive Cancer Center, Keck School of Medicine, University of Southern California, Los Angeles, California, 90033, USA

## Abstract

Deubiquitinase MYSM1 has been shown to play a critical role in hematopoietic cell differentiation and hematopoietic stem cell (HSC) maintenance. Mesenchymal stem cells (MSCs) are multipotent stromal cells within the bone marrow. MSCs are progenitors to osteoblasts, chondrocytes, adipocytes, and myocytes. Although, MSCs have been extensively studied, the roles of MYSM1 in these cells remain unclear. Here we describe the function of MYSM1 on MSC maintenance and differentiation. In this report, we found that *Mysm1−/−* mice had a lower bone mass both in long bone and calvaria compared with their control counterpart. Preosteoblasts from *Mysm*1*−/−* mice did not show changes in proliferation or osteogenesis when compared to WT mice. Conversely, *Mysm1−/−* MSCs showed enhanced autonomous differentiation and accelerated adipogenesis. Our results demonstrate that MYSM1 plays a critical role in MSC maintenance and differentiation. This study also underscores the biological significance of deubiquitinase activity in MSC function. Mysm1 may represent a potential therapeutic target for controlling MSC lineage differentiation, and possibly for the treatment of metabolic bone diseases such as osteoporosis.

Myb-Like, SWIRM, and MPN domains 1(MYSM1) is a histone deubiquitinase that specifically deubiquitinates the histone 2A (H2A) monoubiquitinated at position K119^1^,^2^. H2A K119-monoubiquitination (H2A-K119u) is an abundant chromatin modification associated with transcriptional silencing^1^. Thus MYSM1 reverses gene expression repression at specific loci through its epigenetic regulatory role of transcription^3^,^4^,^5^,^6^,^7^,^8^. Analysis of Mysm1 knockout mice revealed that MYSM1 plays an essential role in the maintenance of hematopoietic stem cell (HSC) quiescence, proper hematopoiesis, and lymphogenesis through controlling the expression of specific transcription factors, including Ebf1^3^, Flt3^4^, Id2^5^, and Gfi1^6^. By using *p53−/− Mysm1−/−* double-deficient mice, some groups uncovered a novel link between Mysm1 deletion and suppression of p53-mediated cell apoptosis during early lymphoid development and other developmental processes^8^,^9^,^10^. In addition, disorders mainly characterized by B alymphocytosis, T lymphopenia, and hematopoiesis impairment caused by MYSM1 deficiency were found in human subjects^11^,^12^. Recently, Panda *et al.* reported that beyond its role in the nucleus, MYSM1 can function in the cytoplasm and acts as a key negative regulator of the innate immune response through inactivation of TRAF3 and TRAF6 complexes^13^.

Bone marrow mesenchymal stem cells (MSCs) are multipotent progenitors of osteoblasts, adipocytes, chondrocytes, and myocytes^14^,^15^,^16^. MSCs are central mediators of bone formation and are involved in the regulation of bone resorption^17^,^18^. Like most stem cells, MSCs use several key transcription regulators to orchestrate their proliferation and lineages choices^19^,^20^,^21^. In spite of the abnormal development of hind limb and tail malformation in Mysm1 deficient mice^3^,^8^,^22^, the function of MYSM1 in MSC proliferation and differentiation remain unclear.

In this study, we analyzed the bone structural phenotype of *Mysm1−/−* deficient mice. Secondly, we isolated pre-osteoblasts and MSCs from both *Mysm1−/−* deficient mice and WT mice, and compared their ability to differentiation and proliferation under specified conditions. We found that MYSM1 is critical for MSC maintenance and proper differentiation to adipocyte or osteoblast.

## Results

### *Mysm1* deficiency results in decreased size and density of mouse long bones

**Figure 1:**
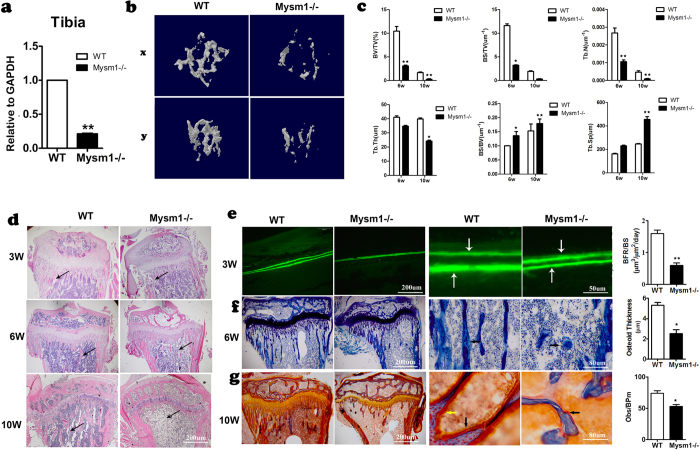
Decreased Bone Mass in long bone *from Mysm1−/−* Mice . (**a**) qRT-PCR of mRNA from tibia tissues of *Mysm1−/−* mice and WT mice, n = 5. (**b**) Micro CT analysis of the sectioned longitudinal sections and cross sections of the distal ends of tibias, n = 7. (**c**) Quantification of trabecular bone fraction. Bone volume/ total volume (BV/TV), bone surface/total volume(BS/TV), trabecular bone number(Tb.N), trabecular thickness (Tb. Th), bone surface/bone volume(BS/BV), and trabecular bone space(Tb.Sp), 6-week and 10-week old mice. n = 4. (**d**) H&E staining of longitudinal sections through the tibia at the metaphysis showing the decreased amount of bone trabeculae (arrows) present in 3-week, 6-week and 10-week old Mysm1*−/−* mice. n = 3. (**e**) Fluorescent micrographs of the double labeled mineralization fronts at the mid-diaphysis of the tibias of 3-week-old Mysm1*−/−* and WT littermate mice. The brackets between the two labeling, tetracyclin at the top and calcein at the bottom indicate the amount of newly formed bone. Scale bar, 200 μm and 50 μm, respectively. The right panel is the bone formation rates (BFRs) of 3-week-old Mysm1*−/−* and WT littermate mice, n = 4. (**f**) Longitudinal sections through the tibia at the metaphysis stained with toluidine blue of 6-week-old Mysm1*−/−* and WT littermate mice; showing the decreased amount of bone trabeculae (arrows) present in the Mysm1*−/−* mice. Scale bar, 200 μm and 80 μm, respectively. Right panel is the osteoid thickness of 6-week-old Mysm1*−/−* and WT littermate mice, n = 5. (**g**) Longitudinal sections through the tibia at the metaphysis stained with golden staining of 10-week-old Mysm1*−/−* and WT littermate mice; showing the decreased amount of osteoblasts (stained with yellow, yellow arrow) and the amount of the osteoid (stained with red, black arrow) present in the Mysm1*−/−* and WT littermate mice. Scale bar, 200 μm and 80 μm, respectively. Right panel is the osteoblasts number of 10-week-old Mysm1*−/−* and WT littermate mice, n = 6. Error bar is ± SEM; *p < 0.05; **p < 0.01.

MYSM1 has been shown to play critical roles in hematopoiesis and lymphogenesis^3^,^4^,^5^,^6^,^7^,^8^,^9^,^10^,^11^,^12^,^13^,^22^,^23^. Our data and those of others have shown that *Mysm1* deficiency in mice results in tail truncation and growth retardation^3^,^8^,^22^. To investigate the physiological role of MYSM1 in bone formation, we systematically analyzed the bones of *Mysm1−/−* mice. *Mysm1−/−* mice and their counterparts were analyzed at 3,6, and 10 weeks of age. The *Mysm1−/−* mice not only had shorter long bones when compared with littermate controls, but also showed a marked decrease in radiodensity within the bone marrow cavity (data not shown). As shown in Fig. 1a, *Mysm1* gene expression, determined by qRT-PCR, was drastically down regulated in tibia tissues of *Mysm1−/−* mice. Three-dimensional reconstruction of the tibia from 10-week-old mice using micro computed tomography (μCT) revealed that *Mysm1−/−* mice were osteoporotic, displaying abruptly decreased tissue bone mass compared to their littermates (Fig. 1b). The bone volume per trabecular volume (BV/TV) and trabecular number (Tb.N), were also lower in Mysm1*−/−* mouse tibia (Fig. 1c).

Bone histomorphometric analysis of the longitudinal sections and cross sections of the distal ends of femurs revealed that *Mysm1−/−* mice had a dramatic decrease in both the number and volume of trabecular bones (Fig. 1d). The difference became more obvious in elder mice. The decreased amount of trabecular bone and thinner cortices confirmed the existence of osteopenia in the *Mysm1−/−* mice.

For assessment of dynamic histomorphometric indices, *in vivo* calcein double-labeling was performed in tibia and calvaria metaphysis. The results suggested that *Mysm1−/−* mice consistently showed a reduction in the mineral apposition rate and bone formation rate at the periosteal surface (Fig. 1e). At high magnification, toluidine blue staining of longitudinal sections of tibia showed that internal osteoblasts had the same morphology in WT and *Mysm1−/−* mice. However, the thickness of the osteoid layer was significantly thinner in *Mysm1−/−* mice (Fig. 1f). Golden staining of 10-week-old *Mysm1−/−* mice (the black arrow denotes the osteoid bone and the yellow arrow denotes the osteoblasts) confirmed the significant decrease of the new and immature osteoid bone, which would support the low bone mass being due to decreased osteoblastic number (Fig. 1g).

### *Mysm1* deficiency affects calvaria bone formation and transcriptional patterns of characterized adipocyte and osteoblast differentiation genes

**Figure 2:**
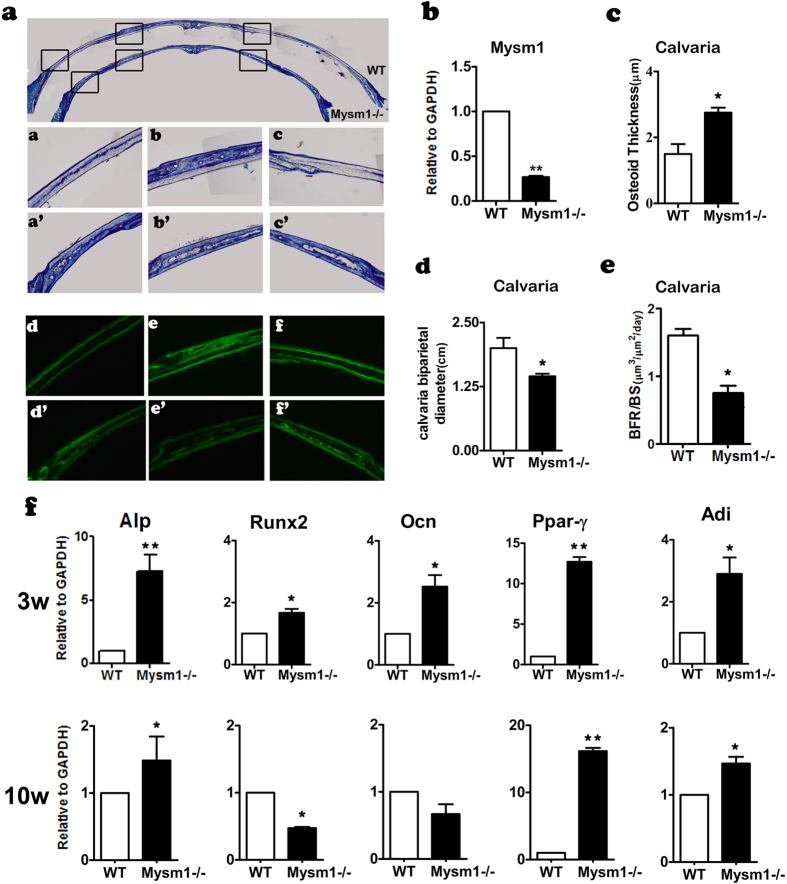
Decreased Bone Mass in calvaria *from Mysm1−/−* Mice. (**a**) Coronal sections of the calvaria stained with toluidine blue and double calcein labeling of 2 week old *Mysm1−/−* mice and WT littermate; Scale bar, 200 μm n = 5. (**b**) qRT-PCR to measure mRNA from calvaria tissues, n = 5. (**c**) The amount of bone Osteoid present in calvaria was increased in 3-week old *Mysm1−/−* mice, compared with WT littermate mice. n = 5. (**d**) The calvaria bi-parietal diameter of *Mysm1−/−* mice is much shorter than that of WT littermate mice, n = 5. (**e**) The bone formation rates (BFRs) of 3-week old Mysm1*−/−* mice is much lower than those of WT littermate mice, n = 5. (**f**) qRT-PCR to measure Alp, Runx2, Ocn, Ppar-γ, Adi mRNA levels in calvaria tissues from 3-week and 10-week old Mysm1*−/−* mice, compared with WT littermate mice (set as 1), n = 5. Error bar is ± SEM; *p < 0.05; **p < 0.01.

Periosteal ossification is the only type of bone formation in calvaria. Therefore, our objective was to study the role of MYSM1 in this process, which would also exclude the effects of bone marrow on bone formation. As in tibia tissue, *Mysm1* expression in the calvaria tissue was also significantly decreased in *Mysm1−/−* mice compared with WT mice (Fig. 2b). Histomorphometrical analysis on the calvaria of 2-week-old mice showed that the diameter of the calvaria in *Mysm1−/−* mice was smaller (Fig. 2a,d).The thickness of the calvaria at the examined position within age matched mice was decreased within the *Mysm1−/−* mice compared to WT controls (Fig. 2e).At high magnification, toluidine blue staining of longitudinal sections of calvaria showed that the osteoblasts had the same morphology in WT and *Mysm1−/−* mice. However, the thickness of the osteoid layer was significantly thicker in *Mysm1−/−* mice, despite the WT mice having formed mature bone in the calvaria (Fig. 2a,c). Interestingly, the calvaria of *Mysm1−/−* mice were scattered with holes, a phenomenon indicative of decreased bone mass (Fig. 2a). To determine the underlying cellular and molecular mechanism of the observed phenotype, qRT-PCR of total RNA from calvaria of 3-week-old and 10-week-old mice was performed. As shown in Fig. 2f, there were significant increases in osteoblast and adipocyte differentiation marker expression in calvaria samples from 3-week-old *Mysm1−/−* mice. At 10 weeks of age calvaria from *Mysm1−/−* mice displayed an increase in the *Alp* osteoblast gene, albeit, not as large as that observed at the 3 week time point. The adipocyte marker Ppar-γ showed a similar increase in expression level at both 10 and 3 weeks within the *Mysm1−/−* mice.

### Effect of *Mysm1* deficiency on pre-osteoblast proliferation and osteogenic differentiation

**Figure 3:**
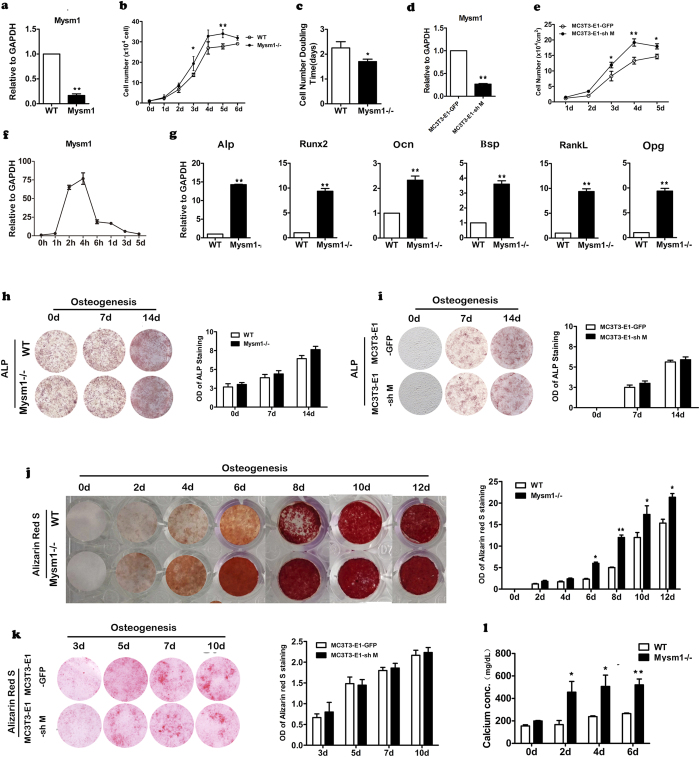
Effect of Mysm1 deficiency on preosteoblast proliferation and osteogenesis. (**a**) qRT-PCR to measure mRNA levels within preosteoblasts from *Mysm1−/−* mice and WT mice, n = 5. (**b**) The cell proliferation curve of primary preosteoblasts from calvaria tissues, n = 4. (**c**) Graph showing the doubling time of primary preosteoblasts, n = 5. (**d**) qRT-PCR to measure mRNA levels of *Mysm1* in MC3T3-E1 cell lines post transduction with LV-GFP (MC3T3-E1-GFP) or LV-shMysm1 (MC3T3-E1-shM), n = 5. (**e**) The cell proliferation curve of MC3T3-E1-GFP and MC3T3-E1-shM. (**f**) qRT-PCR to measure mRNA levels of *Mysm1* in preosteoblast from WT mice, cultured in osteogenic medium, n = 5. (**g**) qRT-PCR to measure mRNA expression of Alp, Runx2, Ocn, Bsp, RankL and Opg. (**h**) Alkaline phosphatase staining (left) and the OD value (right) of *Mysm1−/−* mice and WT littermate MSCs at different time points during *in vitro* osteogenesis. n = 4. (**i**) Alkaline phosphatase staining (left) and the OD value (right) of MC3T3-E1-GFP and MC3T3-E1-shM cells at different time points during *in vitro* osteogenesis. n = 4. (**j**) Alizarin red S staining (left) and the OD value (right) of *Mysm1−/−* mice and WT littermate MSCs at different time points (0–12 days) during *in vitro* osteogenesis. n = 4. (**k**) Alizarin red S staining (left) and the OD value (right) of MC3T3-E1-GFP and MC3T3-E1-shM cells at different time points during *in vitro* osteogenesis. n = 4. (**l**) Calcium amount in MSCs at different time points (0, 2, 4, and 6 days) during *in vitro* osteogenesis, n = 5. Error bar is ± SEM; *p < 0.05; **p < 0.01.

Our data indicates that MYSM1 is essential for normal bone formation. We therefore sought to determine what functional properties of MYSM1 are important to the process of bone formation at a cellular level. We first confirmed that *Mysm1* mRNA expression in pre-osteoblasts from *Mysm1−/−* mice was drastically reduced, here by 80% (Fig. 3a). Secondly, the growth curve and cell doubling time of the pre-osteoblasts obtained from calvaria of the *Mysm1−/−* mice and the WT littermates was determined. As shown in Fig. 3b,c, pre-osteoblasts from *Mysm1−/−* mice had a decreased doubling time and there was an increase in cell number at all-time points assessed. The MC3T3-E1 cell line is a commonly used pre-osteoblast cell line established from newborn C57BL/6 mice. To further understand the growth kinetics of pre-osteoblasts with markedly decreased levels of *Mysm1*, we generated lentiviruses expressing *Mysm1* shRNA or control shRNA, and used these constructs to transduce MC3T3-E1 cells. The knockdown of *Mysm1* was confirmed using qRT-PCR (Fig. 3d). As shown in Fig. 3e, MC3T3-E1 cells transduced with lentiviral vectors targeting *Mysm1* proliferated faster than cells transduced with control lentivirus. *Mysm1* mRNA levels gradually increased in WT pre-osteoblasts during osteoblast differentiation and peaked at 4 hours post induction, then dropped to the lowest point on day5 (Fig. 3f). These data led us to hypothesize that MYSM1 affects osteogenic differentiation at least in regulating the conversion of pro-osteoblasts into osteocytes. Primary pre-osteoblasts were isolated from *Mysm1−/−* mice and controls to assess the expression patterns of bone related genes by qRT-PCR. As demonstrated in Fig. 3g, *Mysm1* deficient primary pre-osteoblasts showed higher Alp, Runx2, Ocn, and Bsp gene expression. The increased propensity of *Mysm1* deficient pre-osteoblast cells to undergo phenotypic changes was further confirmed by culturing these cells under conditions that promote osteogenic differentiation for 2weeks. Alkaline phosphatase (ALP) staining, Alizarin Red S staining, and calcium measurement data (Fig. 3h,j,l) showed that knockdown of *Mysm1* in pre-osteoblasts resulted in higher levels of Alizarin red S staining and indicated faster maturation of osteogenic cells. Furthermore, MC3T3-E1 cells transduced with lentiviruses expressing *Mysm1* shRNA also exhibited enhanced osteoblast differentiation and mineralization after growth in osteogenic induction medium (Fig. 3i,k).

### MSCs from *Mysm1* deficient mice showed increased capacity to autonomous differentiation

**Figure 4:**
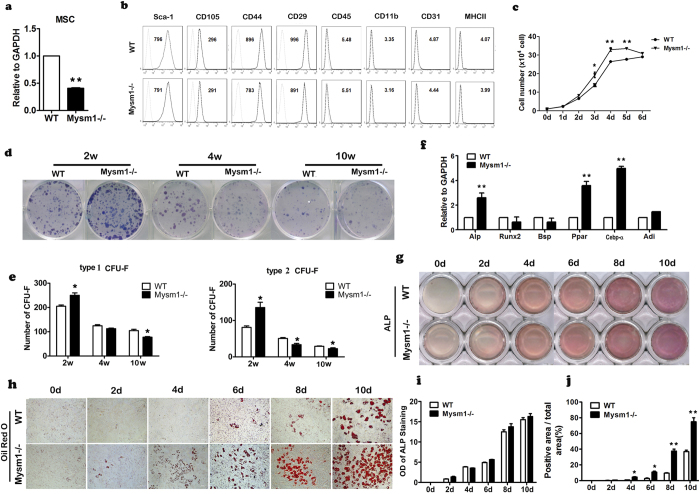
*Mysm1−/−* mice showed increased autonomous differentiation. (**a**) qRT-PCR to measure mRNA levels of *Mysm1* in MSCs, n = 5. (**b**) Flow cytometry analysis of MSCs from *Mysm1−/−* and WT mice. (**c**) The cell proliferation curve of MSCs from tibia and femur tissues, showing that *Mysm1−/−* MSCs have a higher proliferation rate, n = 4. (**d**) Colony-forming unit (CFU) assays showing total colonies formed by *Mysm1−/−* and WT bone marrow stromal cells. Colonies were stained with Giemsa. (**e**) Statistical analyses of CFU assays. The number of colonies (each containing a minimum of 50 cells for type 1 and 100 cells for type 2) was calculated after the total number of *Mysm1−/−* colonies was set to 100%, n = 3. (**f**) qRT-PCR to measure mRNA levels of Alp, Runx2, BSP, Ppar-γ, Cebp-α and Adi of MSCs cultured to confluence for 5 days. (**g**) Alkaline phosphatase (ALP) staining of WT and *Mysm1−/−* MSCs at different time points during *in vitro* culture in α-MEM containing 10% FBS. (**h**) Oil-Red-O staining of WT and *Mysm1−/−* MSCs at different time points during *in vitro* culture in α-MEM containing 10% FBS. (**i**) OD value of ALP staining of WT and *Mysm1−/−* MSCs at different time points during *in vitro* culture in α-MEM containing 10% FBS, n = 4. (**j**) The percentage of Oil-Red-O positive area to the total area (%) of MSCs, at different time points during *in vitro* culture in α-MEM containing 10% FBS, n = 4. Error bar is ± SEM; *p < 0.05; **p < 0.01.

Enhanced osteogenic differentiation of pre-osteoblasts, along with higher expression of RankL and Opg in calvaria from *Mysm1−/−* mice indicating increased osteoblast numbers and total activity, are in contrast to the marked reduction of bone mass in *Mysm1−/−* mice. MSCs give rise to osteoblasts and are essential for bone formation, which prompted us to determine whether the reduction of total MSC number and/or their dysfunction were responsible for this phenotype. MSCs were isolated from the bone marrow of both *Mysm1−/−* mice and control littermates. The reduced expression of *Mysm1* was confirmed by qRT-PCR (Fig. 4a). FACS analysis data showed that MSCs from *Mysm1* deficient mice and their control littermates were similar, both being positive for Sca-1, CD105, CD44, CD29 and negative for CD45, CD11b, CD31, and MHC II markers (Fig. 4b). Similar to what we observed for pre-osteoblasts, MSCs from *Mysm1−/−* mice also exhibited faster growth rates than controls (Fig. 4c). To further assess the proliferation and self-renew potential of these MSCs, colony-forming unit-fibroblastassays (CFU-F) were performed by using MSCs isolated from 2, 4, and 10 week old *Mysm1−/−* mice and WT littermates. Specifically, the MSCs were cultured in stem cell culture medium and the number and size of cell colonies were measured. We scored colonies with more than 50 small, round, or spindle-shaped cells in direct contact with each other as type 1 CFU-F. Clusters with more than 100 cells, including large cells with multiple processes, were classified as type 2 CFU-F. The CFU-F assay data showed that compared with those from WT mice, the numbers of both type 1 and type 2 CFU-F colonies were significantly higher in MSC cultures from 2-week-old *Mysm1−/−* mice, but lower in cultures from 4 and 10 week old *Mysm1−/−* mice compared to controls (Fig. 4d,e).

To further understand MYSM1 function on MSC self-renewal, MSCs from the tibia of *Mysm1−/−* mice and WT mice were seeded in normal culture medium. On the 5th day following cell confluence, the MSCs were stained with ALP and Oil-Red-O, and qRT-PCR was also performed to examine the expression levels of Alp, Runx2, Bsp, Ppar-γ, Cebp-α, and Adi. The results show that Alp, Ppar-γ, Cebp-α, and Adi genes expression levels were increased by 2.5, 3.7, 5, and 1.7 fold respectively in *Mysm1−/−* cells compared to WT cells (Fig. 4f). ALP is considered as an important earlier osteoblast marker gene. The results demonstrated that *Mysm1* deficient bone marrow stromal cells had higher ALP expression levels than WT cells. These data suggest that osteoblast differentiation occurred earlier in *Mysm1* deficient cells than in WT cells under basic culture conditions (Fig. 4g). In addition, the pattern of ALP expression levels in *Mysm1−/−* MSCs may relate to their unique proliferation capacity, since ALP levels in bone cells are associated with cell cycle regulation^24^. When *Mysm1* deficient MSCs were stained with Oil Red O, there were dramatic increased numbers of adipocytes than that of WT MSC (Fig. 4h–j).

### *Mysm1* deficient MSCs display enhanced adipogenic differentiation

**Figure 5:**
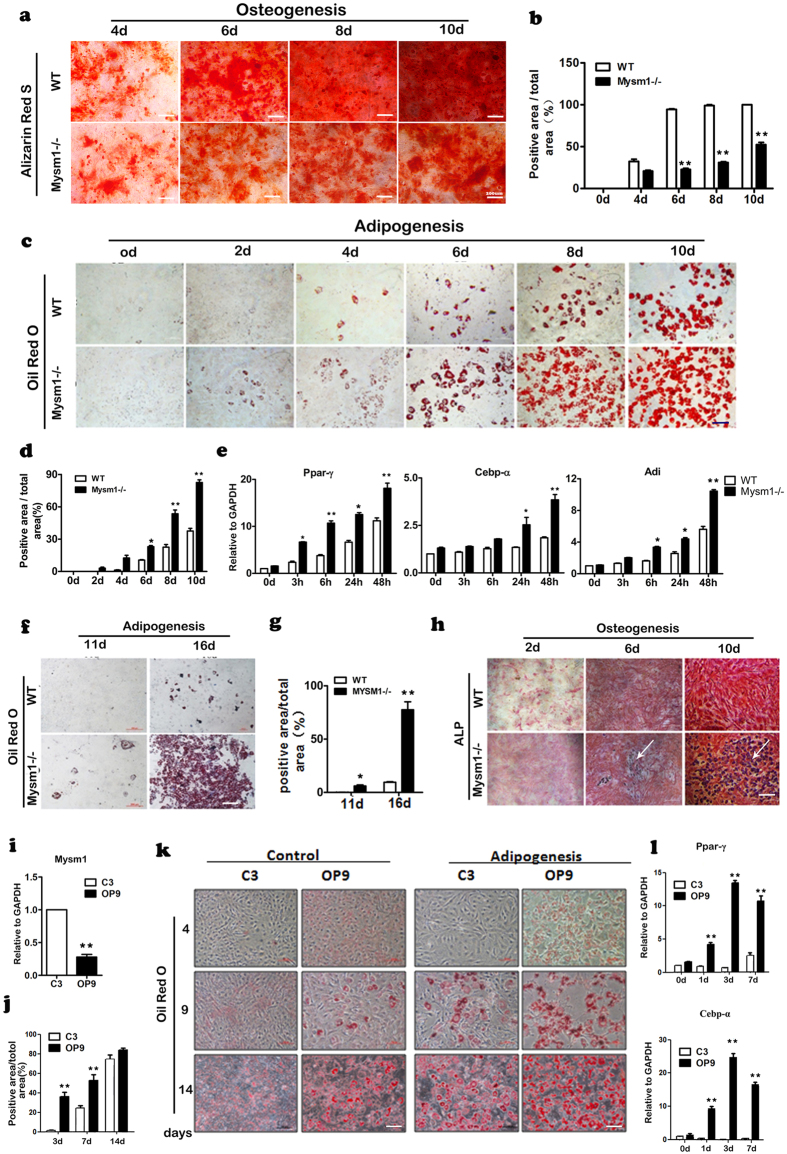
*Mysm1−/−* MSCs show enhanced adipocyte differentiation. (**a**) Alizarin red staining of WT and *Mysm1−/−* MSCs at different time points during *in vitro* osteogenesis. Scale bar, 200 μm. (**b**) Percentage of Alizarin red positive staining area to the total area (%), n = 4. (**c**) Oil-Red-O staining of WT and *Mysm1−/−* MSCs at different time points during *in vitro* adipogenesis, Scale bar, 200 μm. (**d**) The percentage of Oil-Red-O positive area to the total area (%), n = 4. (**e**) qRT- PCR to measure mRNA levels of Ppar-γ, Cebp-α, and Adi in *Mysm1−/−* and WT MSCs at different time points during *in vitro* adipogenesis, n = 5. (**f**) Oil-Red-O staining of preosteoblasts from WT and *Mysm1−/−* mice from calvaria tissues at 11 and 16 days during *in vitro* adipogenesis, Scale bar, 200 μm. (**g**) The percentage of Oil-Red-O positive area to the total area (%), n = 4. (**h**) ALP staining of WT and *Mysm1−/−* preosteoblasts from calvaria tissues at days 2, 6, and 10 during *in vitro* osteogenesis, scale bar, 200 μm. (**i**) qRT-PCR to measure mRNA levels of *Mysm1* in the C3H/10T1/2 and OP9 cell lines, n = 5. (**j,k**) Percentage of Oil-Red-O positive area to the total area (%) and Oil-Red-O staining of the cell lines at different time points during *in vitro* adipogenesis. (**l**) qRT-PCR to measure mRNA levels of Ppar-γ and Cebp-α in the C3H/10T1/2 and OP9 cell lines at different time points during *in vitro* osteogenesis, n = 5. Error bar is ± SEM; *p < 0.05; **p < 0.01.

Because *Mysm1−/−* MSCs displayed properties of increased autonomous differentiation, we further investigated their ability to undergo lineage specific differentiation. To assess the effect of Mysm1 on MSC differentiation into osteoblasts, we performed Alizarin red S staining of the calcium nodules at the different time of osteogenic induction. Alizarin red S is a dye that binds to calcium deposited in the matrix by mature osteoblasts. As shown in Fig. 5a,b, compared with MSCs from WT mice, the size and the positive area of Alizarin red S staining were smaller in *Mysm1−/−* MSC, which indicated the compromised osteoblast differentiation capacities.

MSCs are the precursors of adipocytes, and will give rise to these cells under specific induction medium. When *Mysm1−/−* MSCs were stained with Oil-Red-O, a dramatic increase in adipocytes was observed. We also found that *Mysm1−/−* MSCs differentiated into mature adipocytes much faster than WT MSCs because Oil-Red-O staining was positive within 2–4 days for differentiating *Mysm1−/−* MSCs versus 4–6 days for differentiating WT MSCs (Fig. 5c). Additionally, the *Mysm1−/−* MSC oil drops were much larger than those of WT MSCs, suggesting that *Mysm1−/−* MSCs have a higher tendency to develop into adipocytes than WT MSCs (Fig. 5c,d). Using qRT-PCR, we observed that the Ppar-γ, Cebpα, and Adi mRNA levels gradually increased in *Mysm1* deficient MSCs during adipogenic differentiation and peaked at 48 hours upon differentiation (Fig. 5e). When cultured under basic conditions (α-MEM plus 10% FBS), the cells cultured from calvaria tissue showed characteristics of pre-osteoblasts and osteoblasts. However, when cells were cultured in adipogenic differentiation medium, the *Mysm1−/−* cells stained positive for Oil-Red-O on the 11th day and formed very large adipocytes by the16th day, while the WT group showed only few Oil-Red-O positive cells (Fig. 5f,g). Surprisingly, when the *Mysm1−/−* MSCs were cultured in osteogenic differentiation medium, cells also stained positive for Oil-Red-O, however, this was not observed in WT cells (Fig. 5h). The enhanced adipocyte differentiation phenomena were also observed in established MSC lines. C3H/10T1/2 cells and OP9 cells are commonly used in MSC related studies, and we found that OP9 cells showed lower *Mysm1* expression than C3H/10T1/2 cells (Fig. 5i). Both cell lines were cultured in adipogenic induction medium, then Oil-Red-O staining and qRT-PCR examining Ppar-γ and Cebpα expression were performed at different time points during induction. The results demonstrated that OP9 cells with lower *Mysm1* expression showed enhanced adipogenic differentiation (Fig. 5j–l).

## Discussion

In this study we provide evidence that MYSM1 function is essential for normal bone formation and MSC differentiation. *Mysm1−/−* mice show characteristic osteopenia, a condition that became more severe with age. MSCs with low *Mysm1* expression show uncontrolled autonomous differentiation and a propensity to adipocyte differentiation.

We and others have found that MYSM1 enzymatic functions are crucial for bone marrow hematopoiesis and lymphocyte differentiation in mice^3^,^4^,^5^,^6^,^7^,^8^,^9^,^10^,^11^,^12^,^13^,^22^,^23^. Although defects in cellular interactions, bone marrow microenvironment, and differentiation potential, the bone marrow niche can be presumed to affect normal bone formation^25^,^26^. However, Mysm1^fl/fl^:Tie2-cre mice with hematopoietic dysfunction did not show bone and tail malformation (data not shown). In addition, Mysm1 ^fl/fl^:LysM-cre mice with ablated MYSM1 in macrophages were also lack of tail malformation^13^.

An osteoporotic phenotype may result from either decreased osteoblastic bone formation or/and increased osteoclastic bone resorption. Our *in vivo* data showed that the bone formation rates and osteoblast numbers were decreased in *Mysm1−/−* mice. However, *in vitro,* the committed osteoblastic cells from *Mysm1−/−* mice showed enhanced proliferation and differentiation capacities. In addition, *Mysm1* knockdown in MC3T3-E1 cells resulted in enhanced proliferation and differentiation within these cells. Osteoblast differentiation is tightly control by several factors. Surprisingly, the expression levels of *Alp, Runx2, Osterix, Bsp* and *Ocn* bone related genes were increased in 2- and 4-week-old *Mysm1−/−* mice compared with their WT littermates, data not consistent with the decreased osteoblastic bone formation observed by morphologic analysis. The osteoclastic bone resorption related genes-*RankL* and *Opg* were increased significantly in both 2- and 4-week-old *Mysm1* deficient mice. The ratio of RankL/Opg mRNA was an important osteoporotic phenotype^27^. The levels were altered in bones from *Mysm1* deficient mice compared with bone from WT mice. Whether changes in these bone specific genes contributed to the osteopenic phenotype observed *in vivo* requires further in depth analysis between the interactions of these factors with MYSM1.

Quiescence is critical for the maintenance, survival and self-renewal of MSCs^28^. Previously Wang *et al.* reported that MYSM1 plays an essential and intrinsic role in maintaining the quiescence and pool size of HSCs^6^. Within the bone marrow, MSCs are critical for bone homeostasis. Our data demonstrated that MSCs deficient in *Mysm1* display autonomous differentiation, and are espeically prone to form adipocytes, which indicates that Mysm1 is also essential for MSC pool maintenance and differentiation. Published studies of mice deficient in *p21, p53, Gfi1,* or *Pten* have shown that a loss of quiescence and unscheduled MSC proliferation results in the loss of self-renewal ability and leads to stem cell exhaustion^29^,^30^,^31^. Our data from CFU-F assays showed that CFU efficiency of MSCs was decreased significantly in aged *Mysm1* deficient mice. Therefore, stem cell exhaustion of the *Mysm1* deficient MSCs may be a factor in promoting the development of the osteoporotic phenotype. Gatzka *et al.* revealed that MYSM1 regulated hematopoesis through inlfluencing the interplay of factors along the p19ART/p53 axis^8^. Several studies have reported that p53 activity can influence the cell fate specification of MSCs^31^,^32^,^33^. Though p53 expression is also increased in *Mysm1* deficient MSCs, we did not observe an increased percentage of apoptosis in *Mysm1* deficient MSCs (data not shown). Further studies are required to confirm and characterized the interation between Mysm1 and p53 in MSCs.

The altered balance between normal osteogenesis and adipogenesis by MSCs may also contribute to osteoporosis in *Mysm1* deficient mice. The reduction in trabecular bone volume that occurs in *Mysm1* deficient mice is frequently associated with increased adipogenesis. *In vitro* induction assay also demonstrated a reduction in osteogenesis and an enhancement of adipogenesis in *Mysm1* deficient MSCs. The differentiation of MSCs is indicated by a complex network of transcription factors, such as Osterix, Runx2, Cebp-α, Ppar-γ, and signaling components, such as BMP/TGFβ and Wnt pathways^34^,^35^,^36^. Stem cell populations undergo dynamic reprogramming of gene expression profiles during lineage commitment and maturation. More and more studies have reported that miRNA, EzH2, and KDM6A act as epigenetic switches for MSC lineage specification^37^,^38^,^39^. The detailed mechanisms of MYSM1 function on MSC fate decisions need further investigation.

Overall, this study identified a role of deubiquitinase MYSM1 in regulating proliferation and differentiation of MSCs. Furthermore, these changes were shown to directly impact patterns of bone formation within mice. Understanding the mechanism of MYSM1 in MSC regulation of lineage determination is pivotal for understanding bone cell differentiation, under circumstances where a decrease in bone mass result from a qualitative or quantitative alteration in the MSC pool.

## Methods

### Mice

*Mysm1−/−* mice were generated as described previously^3^. In summary, they were generated by crossing Mysm1 mRNA truncation-first floxed mice (Mysm1 tm1a/tm1a). In all experiments, WT littermates (+/+) matched by gender and age were used for controls. Mice were maintained in a pathogen-free barrier facility. All animal experiments were performed according to the “Guide for the Care and Use of Laboratory Animals” approved by Beijing Institute of Basic Medical Sciences. The institutional Ethics Review Committee for Animal Experimentation approved all experimental protocols.

### Isolation and Culture of Calvaria-Derived Cells

Primary pre-osteoblasts were isolated and cultured from calvaria. In summary, calvaria tissues were washed five times in PBS, minced into <1 mm^3^ pieces, treated enzymatically (1 mg/ml Collagenase II (Gibco) in PBS for 60 min at 37 °C, and gently dissociated by pipetting. Cells and bone chips were then cultured in α-MEM supplemented with 10% fetal bovine serum (FBS, Gibco); 100 U/ml penicillin; 100 U/ml streptomycin; 2.5 μg/ml ascorbic acid (Sigma, St. Louis,MO) and 5 ml Glutamax, after washed by PBS for three times, under 37 °C at 5% CO_2_ condition.The living cells were counted in a Malassez hemocytometer after staining with trypan blue and the cell number was calculated on the average of three counts. The attached cells were passaged every other day.

### Isolation and culture of mouse mesenchymal stem cells (MSCs)

MSCs were flashed out from bone cavity of femurs and tibias with 0.5% FBS in PBS. Single-cell suspension of all nuclear cells was obtained by passing through 70 μm cell strainer (BD Bioscience). All nuclear cells were seeded at 1 × 10^7^ into 100 mm culture dishes (Corning) and initially incubated for 48 hours under 37 °C at 5% CO_2_ condition. To eliminate the non-adherent cells, the cultures were washed with PBS twice. The attached cells were passaged every other day. The MSCs were cultured with α-MEM (Invitrogen) supplemented with 10% FBS, 2 mM L-glutamine (Invitrogen), 100 U ml/L penicillin, and 100 μg ml/L streptomycin (Invitrogen).

### Culture of mouse cell lines

Three kinds of cell lines were cultured. Firstly, MC3T3-E1were cultured with α-MEM supplemented with 10% FBS, 2 mM L-glutamine, 100 U ml/L penicillin, and 100 μg ml/L streptomycin (Invitrogen); secondly, C3H/10T1/2 were cultured with α-MEM supplemented with 10% FBS, 2 mM L-glutamine, 100 U ml/L penicillin, and 100 μg ml/L streptomycin (Invitrogen Corporation);thirdly, OP9 were cultured withα-MEM supplemented with 20% FBS, 2 mM L-glutamine, 100 U ml/L penicillin, and 100 μg ml/L streptomycin (Invitrogen).

### *In vitro* differentiation assay

For *in vitro* osteogenesis and adipogenesis, cells were induced with osteogenic induction medium containing 0.1 mM dexamethasone, 50 mM ascorbate-2 phosphate, 10 mM glycerophosphate (Sigma). Cells were maintained in culture for 0, 2, 4, 6, 8, 10 days. Cells were stained for alkaline phosphatase (ALP) activity using alkaline phosphatase kit (Sigma). ALP activity was determined using p-nitrophenylphosphate as a substrate in 0.05M 2-amino-2-methyl- propanol and 2 mM MgCl2 (pH 10.5). The amount of p-nitrophenol released was estimated by measuring absorbance at 410 nm. Protein concentration was determined using a BCA protein assay kit (Pierce Chemical Co., Rockford, IL), and fixed with 4% PFA for 30 min, and then were stained with 1% Alizarin red S (Sigma, St. Louis, MO) for 1 h at 37 °C. Amounts of calcium secreted were measured by using a Calcium colormetric assay kit 380–250 (Biomedical Technologies Inc., Stoughton, MA). To induce adipogenic differentiation, cells were cultured in adipogenic induction medium containing 1 mM dexamethasone, 200 mM indomethacin, 0.5 mM 3-isobutyl-1-methyl-xanthine and 10 μg/mL insulin (Sigma). Cells were stained for fat droplets using the Oil-Red-O (Sigma).

### Lentivirus production and transduction

Recombinant lentiviral vectors (LV-shMysm1 and LV-GFP) were produced and transduced as described in our previous publications^7^.

### Calcein staining

To analyze the changes in bone formation rates (BFRs), double fluorescence labeling was performed and analyzed in tibial and calvaria metaphysis, we injected calcein (Sigma) intraperitoneally at 7.5 mg/kg on days 10 and 2 before killing, and bone tissues were removed and fixed in 70% ethanol for 48 h. The specimens were dehydrated through a graded series of ethanol (70–100%) and embedded in methylmethacrylate without prior decalcification. 7-μm sections were cut using a Leica 2165 rotary microtome, and the unstained sections were viewed using fluorescence microscopy, and the following dynamic indices of bone formation were measured^40^: (a) Labeled bone surface (BS) or mineralizing surface; (b) Mineral appositional rate (m/day)mean distance between two fluorescent labels divided by the number of days between labels; and (c) BFR/BS,m3/m 2/day mineralizing surface mineral appositional rate/BS.

### Toluidine blue staining

Undecalcified bones were embedded in methylmethacrylate, and 5-μm sections were prepared on a rotation microtome (Jung, Heidelberg, Germany) as described previously^41^,^42^. Sections were stained with 1% toluidine blue, or von Kossa reagent (3% silver nitrate counterstained with Kernechtrot), or hematoxylin/eosin, and evaluated using a Zeiss microscope (Carl Zeiss, Jena, Germany). Histomorphometrical analysis was performed on tibiae and vertebrae according to the American Society for Bone and Mineral Research (ASBMR) standards^43^ using the OsteoMeasure Analysis System (Osteometrix, Atlanta, GA). Statistical differences between groups were assessed by Student’s t-test.

### Micro computed tomography (μCT)

The tibiae were scanned using a micro CT scanner (model 1172, Skyscan, Aartselaar, Belgium) at 50 Kv and 200 μA with a 0.5 aluminium filter using a detection pixel size of 4.3 μm. Images were captured every 0.7° through 180° rotation of the bone. The scanned images were reconstructed using the Skyscan Recon software and analysed using Skyscan CT analysis software. A standard trabecular bone volume of interest was chosen starting 0.2 mm from the growth plate and included all the trabeculae in 1 mm^3^ of bone.

### qRT-PCR

Total RNA was extracted with TRIZOL (Sigma) and reverse transcribed into cDNA with reverse transcriptase kit (Takara). cDNA was used as template in qRT-PCR with SYBR Green reagent from TOYOBO (Shanghai, China) to determine specific gene expression. Primers are available upon request.

### Flow cytometric analysis

For surface molecule staining, cells were harvested using 0.25% trypsin, and stained for 30 min at 4 °C. Antibodies against mouse Sca-1, CD45, CD105, CD44, MHC II, CD11b, CD29, and CD31were purchased from BioLegend (San Diego, CA, USA). After washing 3 times in PBS, cells were fixed in 1% paraformaldehyde. Data were collected by a BD FACSCalibur (BD Biosciences, San Jose, CA, USA) and analyzed with FlowJo software 7.6 (TreeStar, Ashland, OR, USA).

### Statistical Analysis

All data were analyzed using Prism 5.0 software and are presented as mean ± SEM. Statistical significance was assessed by unpaired two-tailed Student’s t-tests.

## Additional Information

**How to cite this article**: Li, P. *et al.* Deubiquitinase MYSM1 Is Essential for Normal Bone Formation and Mesenchymal Stem Cell Differentiation. *Sci. Rep.* **6**, 22211; doi: 10.1038/srep22211 (2016).
